# Bis[2-(2-amino­eth­yl)-1*H*-benzimidazole-κ^2^ *N* ^2^,*N* ^3^](nitrato-κ^2^ *O*,*O*′)cobalt(II) chloride trihydrate

**DOI:** 10.1107/S1600536812018612

**Published:** 2012-05-02

**Authors:** Jing Zhao, Heng Zhang, Guoyi Zhu

**Affiliations:** a Changchun Institute of Applied Chemistry, Chinese Academy of Sciences, Changchun 130022, People’s Republic of China; b Graduate University of Chinese Academy of Sciences, Beijing 100049, People’s Republic of China; c The Experimental and Practical Teaching Centre, Shijiazhuang University of Economics, Shijiazhuang 050031, People’s Republic of China; d Instrumental Analysis Center, Hebei Normal University, Shijiazhuang 050024, People’s Republic of China

## Abstract

In the title compound, [Co(NO_3_)(C_9_H_11_N_3_)_2_]Cl·3H_2_O, the Co^II^ atom is coordinated by four N atoms from two chelating 2-(2-amino­eth­yl)-1*H*-benzimidazole ligands and two O atoms from one nitrate anion in a distorted octa­hedral coordination environment. In the crystal, N—H⋯Cl, N—H⋯O, O—H⋯Cl and O—H⋯O hydrogen bonds link the complex cations, chloride anions and solvent water mol­ecules into a three-dimensional network. π–π inter­actions between the imidazole and benzene rings and between the benzene rings are observed [centroid–centroid distances = 3.903 (3), 3.720 (3), 3.774 (3) and 3.926 (3) Å].

## Related literature

For background to the coordination chemistry of benzimidazole and 2-substituted benzimidazole derivatives towards transition metal ions, see: Téllez *et al.* (2008 ▶). For the structures and properties of transition metal complexes with 2-(2-amino­eth­yl)benzimidazole, see: Dash *et al.* (1995 ▶); Zhang *et al.* (2008 ▶). For the synthesis of the 2-(2-amino­eth­yl)benz­imid­azole ligand, see: Cescon & Day (1962 ▶).
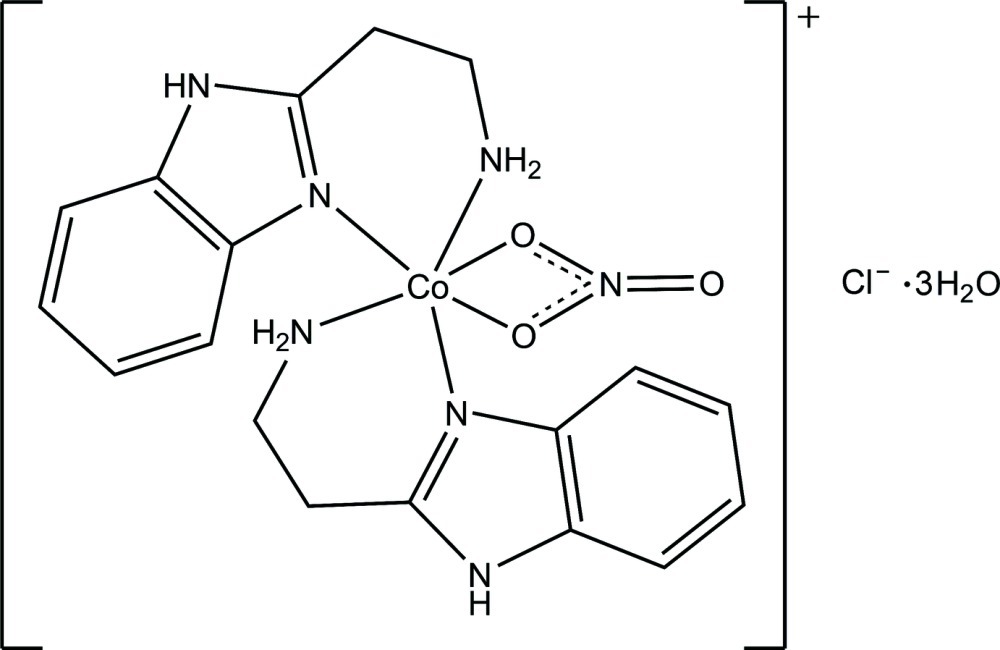


## Experimental

### 

#### Crystal data

[Co(NO_3_)(C_9_H_11_N_3_)_2_]Cl·3H_2_O
*M* *_r_* = 532.85
Triclinic, 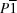

*a* = 7.408 (2) Å
*b* = 9.808 (3) Å
*c* = 17.280 (6) Å
α = 76.238 (7)°
β = 89.203 (7)°
γ = 67.867 (5)°
*V* = 1125.6 (6) Å^3^
*Z* = 2
Mo *K*α radiation
μ = 0.93 mm^−1^
*T* = 296 K
0.28 × 0.27 × 0.26 mm

#### Data collection

Bruker APEXII CCD diffractometer
Absorption correction: multi-scan (*SADABS*; Sheldrick, 1996 ▶) *T*_min_ = 0.780, *T*_max_ = 0.794
5677 measured reflections
3936 independent reflections
2895 reflections with *I* > 2σ(*I*)
*R*_int_ = 0.029

#### Refinement

*R*[*F* ^2^ > 2σ(*F* ^2^)] = 0.050
*wR*(*F* ^2^) = 0.131
*S* = 0.99
3936 reflections
298 parameters
3 restraints
H-atom parameters constrained
Δρ_max_ = 0.35 e Å^−3^
Δρ_min_ = −0.86 e Å^−3^

### 

Data collection: *APEX2* (Bruker, 2007 ▶); cell refinement: *SAINT* (Bruker, 2007 ▶); data reduction: *SAINT*; program(s) used to solve structure: *SHELXS97* (Sheldrick, 2008 ▶); program(s) used to refine structure: *SHELXL97* (Sheldrick, 2008 ▶); molecular graphics: *SHELXTL* (Sheldrick, 2008 ▶); software used to prepare material for publication: *SHELXTL*.

## Supplementary Material

Crystal structure: contains datablock(s) I, global. DOI: 10.1107/S1600536812018612/hy2542sup1.cif

Structure factors: contains datablock(s) I. DOI: 10.1107/S1600536812018612/hy2542Isup2.hkl

Additional supplementary materials:  crystallographic information; 3D view; checkCIF report

## Figures and Tables

**Table 1:** Hydrogen-bond geometry (Å, °)

*D*—H⋯*A*	*D*—H	H⋯*A*	*D*⋯*A*	*D*—H⋯*A*
N2—H2⋯O4	0.86	1.91	2.757 (5)	168
N3—H3*B*⋯Cl1^i^	0.90	2.56	3.322 (3)	143
N3—H3*C*⋯O3^ii^	0.90	2.08	2.897 (4)	150
N5—H5⋯Cl1^iii^	0.86	2.29	3.148 (4)	174
N6—H6*A*⋯O4^i^	0.90	2.54	3.299 (5)	142
N6—H6*B*⋯O3^ii^	0.90	2.12	2.897 (4)	145
O4—H1*O*4⋯O5	0.85	1.92	2.766 (4)	179
O4—H2*O*4⋯O6^iv^	0.85	1.93	2.781 (5)	179
O5—H1*O*5⋯Cl1^v^	0.85	2.41	3.189 (3)	153
O5—H2*O*5⋯O1^vi^	0.85	2.03	2.872 (4)	174
O6—H1*O*6⋯O2	0.85	2.01	2.828 (4)	162
O6—H2*O*6⋯Cl1^vi^	0.85	2.34	3.192 (4)	176

Symmetry codes: (i) 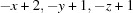; (ii) 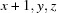; (iii) 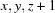; (iv) 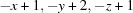; (v) 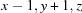; (vi) 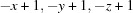.

**Fig. 1.**
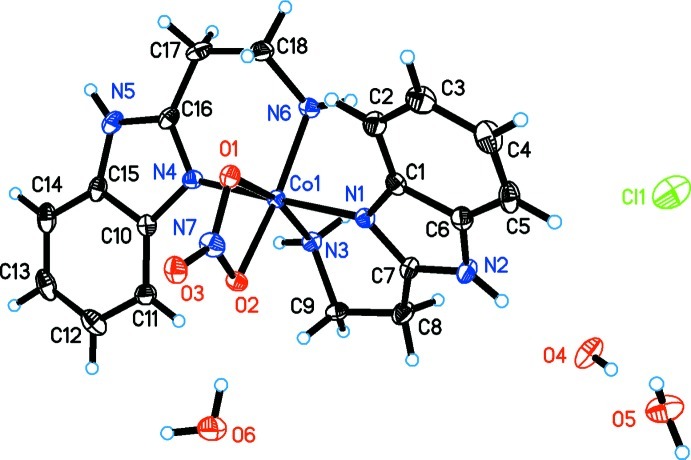
Molecular structure of the title complex, with displacement ellipsoids drawn at the 30% probability level.

**Fig. 2.**
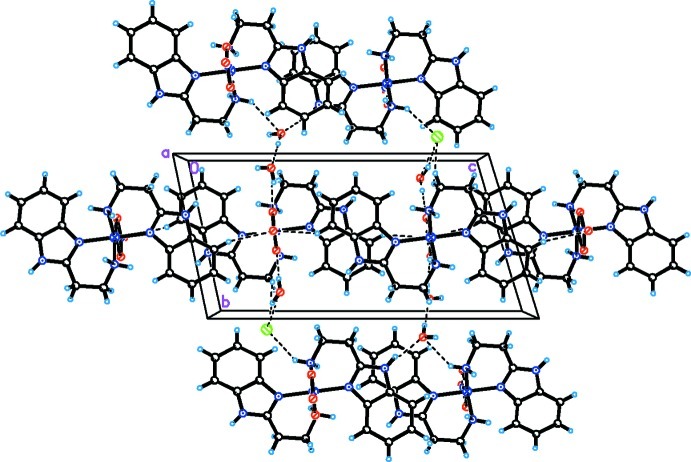
The packing diagram viewed along the a axis. Dashed lines denote hydrogen bonds.

## supplementary crystallographic information

### Comment

Benzimidazole and 2-substituted benzimidazole derivatives are important heteroaromatic compounds acting as multidentate ligands towards transition metal ions in coordination chemistry (Téllez *et al.*, 2008). 2-(2-Aminoethyl)benzimidazole is a bidentate ligand possessing two aromatic rings and can chelate a 3d transition-metal ion through two N atoms of the pendant aminoethyl group and the imidazole ring (Zhang *et al.*, 2008). Furthermore, this ligand has a larger conjugated π-system than imidazole, which is expected to display a stability-enhancement due to the hydrophobic interaction with the substituted group of the amino acids or to be involved in aromatic ring π–π stacking effects with purine and pyrimidine bases. Based on the inherent characters of the benzimidazole group, a large number of studies have been reported on metal complexes of benzimidazole-based ligands (Dash *et al.*, 1995). To our knowledge, Co^II^ complexes with 2-(2-aminoethyl)benzimidazole ligand have not been reported. In this paper, we report the crystal structure of one such complex.

The title compound consists of a [Co(NO_3_)(C_9_H_11_N_3_)_2_]^+^ complex cation, a chloride anion and three hydrate solvent molecules. As shown in Fig. 1, two bidentate 2-(2-aminoethyl)benzimidazole ligands are coordinated to the Co^II^ atom solely *via* two N atoms. A bidentate nitrate is coordinated to the Co^II^ atom *via* two O atoms. The coordination geometry around the Co^II^ atom is distorted octahedral, with bite angles of 68.37 (12)° for the nitrate anion and 89.44 (13) and 89.37 (14)° for the two bidentate ligands. The other *cis* bond angles at the Co^II^ atom fall in the range of 89.37 (14)–98.11 (13)° and the *trans* bond angles are 166.29 (13), 166.39 (13) and 178.40 (14)°, suggesting a significant deviation from a perfect octahedral coordination. The Co—N bond lengths range from 1.948 (3) to 1.957 (3) Å, with an average of 1.952 (3) Å. The Co—O bond lengths are 1.928 (3) and 1.930 (3) Å. Extensive hydrogen bonds in the crystal, as shown in Fig. 2 and Table 1, link the complex cations, chloride anions and hydrate solvent molecules into a three-dimensional network.

### Experimental

The title compound was prepared by adding a methanol solution (5 ml) of Co(NO_3_)_2_.6H_2_O (0.1 mmol) to a methanol solution (5 ml) of 2-(2-aminoethyl)benzimidazole dihydrochloride (0.2 mmol) neutralized by sodium hydroxide (Cescon & Day, 1962). The mixture was stirred at room temperature for 15 h and then filtered. Purple crystals suitable for X-ray diffraction were obtained by slow evaporation of the solvent after several days. Analysis, calculated for C_18_H_28_ClCoN_7_O_6_: C 40.57, H 5.30, N 18.40%; found: C 40.42, H 5.48, N 18.36%.

### Refinement

H atoms bonded to C and N atoms were positioned geometrically and refined as riding atoms, with C—H = 0.93 (aromatic), 0.97 (CH_2_) and N—H = 0.86 (NH), 0.90 (NH_2_) Å and with *U*_iso_(H) = 1.2*U*_eq_(C,N). H atorms of water molecules were found from a difference Fourier map and refined as riding atoms, with O—H = 0.85 Å and with *U*_iso_(H) = 1.2*U*_eq_(O).

### Crystal data

**Table:**

[Co(NO_3_)(C_9_H_11_N_3_)_2_]Cl·3H_2_O	*Z* = 2
*M**_r_* = 532.85	*F*(000) = 554
Triclinic, *P*1	*D*_x_ = 1.572 Mg m^−^^3^
Hall symbol: -P 1	Mo *K*α radiation, λ = 0.71073 Å
*a* = 7.408 (2) Å	Cell parameters from 1285 reflections
*b* = 9.808 (3) Å	θ = 2.9–23.7°
*c* = 17.280 (6) Å	µ = 0.93 mm^−^^1^
α = 76.238 (7)°	*T* = 296 K
β = 89.203 (7)°	Cube, purple
γ = 67.867 (5)°	0.28 × 0.27 × 0.26 mm
*V* = 1125.6 (6) Å^3^	

### Data collection

**Table:**

Bruker APEXII CCD diffractometer	3936 independent reflections
Radiation source: fine-focus sealed tube	2895 reflections with *I* > 2σ(*I*)
Graphite monochromator	*R*_int_ = 0.029
φ and ω scans	θ_max_ = 25.0°, θ_min_ = 2.3°
Absorption correction: multi-scan (*SADABS*; Sheldrick, 1996)	*h* = −8→8
*T*_min_ = 0.780, *T*_max_ = 0.794	*k* = −9→11
5677 measured reflections	*l* = −20→20

### Refinement

**Table:**

Refinement on *F*^2^	Primary atom site location: structure-invariant direct methods
Least-squares matrix: full	Secondary atom site location: difference Fourier map
*R*[*F*^2^ > 2σ(*F*^2^)] = 0.050	Hydrogen site location: inferred from neighbouring sites
*wR*(*F*^2^) = 0.131	H-atom parameters constrained
*S* = 0.99	*w* = 1/[σ^2^(*F*_o_^2^) + (0.0713*P*)^2^] where *P* = (*F*_o_^2^ + 2*F*_c_^2^)/3
3936 reflections	(Δ/σ)_max_ = 0.001
298 parameters	Δρ_max_ = 0.35 e Å^−^^3^
3 restraints	Δρ_min_ = −0.86 e Å^−^^3^

### Special details

**Table:**

Geometry. All e.s.d.'s (except the e.s.d. in the dihedral angle between two l.s. planes) are estimated using the full covariance matrix. The cell e.s.d.'s are taken into account individually in the estimation of e.s.d.'s in distances, angles and torsion angles; correlations between e.s.d.'s in cell parameters are only used when they are defined by crystal symmetry. An approximate (isotropic) treatment of cell e.s.d.'s is used for estimating e.s.d.'s involving l.s. planes.
Refinement. Refinement of *F*^2^ against ALL reflections. The weighted *R*-factor *wR* and goodness of fit *S* are based on *F*^2^, conventional *R*-factors *R* are based on *F*, with *F* set to zero for negative *F*^2^. The threshold expression of *F*^2^ > σ(*F*^2^) is used only for calculating *R*-factors(gt) *etc*. and is not relevant to the choice of reflections for refinement. *R*-factors based on *F*^2^ are statistically about twice as large as those based on *F*, and *R*- factors based on ALL data will be even larger.

### Fractional atomic coordinates and isotropic or equivalent isotropic displacement parameters (Å^2^)

**Table:**

	*x*	*y*	*z*	*U*_iso_*/*U*_eq_	
Co1	0.76176 (7)	0.51980 (6)	0.74870 (3)	0.01888 (18)	
Cl1	1.08278 (18)	0.11248 (13)	0.14279 (7)	0.0416 (3)	
C1	0.7611 (6)	0.4443 (5)	0.5882 (2)	0.0240 (9)	
C2	0.7801 (6)	0.2936 (5)	0.6108 (3)	0.0324 (10)	
H2A	0.7928	0.2425	0.6644	0.039*	
C3	0.7796 (7)	0.2221 (6)	0.5509 (3)	0.0413 (12)	
H3	0.7923	0.1211	0.5646	0.050*	
C4	0.7604 (7)	0.2986 (6)	0.4698 (3)	0.0430 (12)	
H4	0.7585	0.2476	0.4310	0.052*	
C5	0.7445 (6)	0.4455 (6)	0.4467 (3)	0.0371 (11)	
H5A	0.7321	0.4959	0.3929	0.045*	
C6	0.7475 (6)	0.5175 (5)	0.5072 (2)	0.0291 (10)	
C7	0.7441 (6)	0.6755 (5)	0.5797 (2)	0.0248 (9)	
C8	0.7469 (7)	0.8163 (5)	0.5963 (3)	0.0361 (11)	
H8A	0.6403	0.9022	0.5633	0.043*	
H8B	0.8678	0.8260	0.5794	0.043*	
C9	0.7298 (6)	0.8281 (5)	0.6815 (2)	0.0285 (10)	
H9A	0.7666	0.9102	0.6877	0.034*	
H9B	0.5949	0.8524	0.6939	0.034*	
C10	0.6666 (6)	0.5948 (5)	0.9090 (2)	0.0239 (9)	
C11	0.5304 (6)	0.7426 (5)	0.8863 (2)	0.0317 (10)	
H11	0.4912	0.7924	0.8328	0.038*	
C12	0.4541 (7)	0.8140 (6)	0.9470 (3)	0.0439 (13)	
H12	0.3620	0.9134	0.9332	0.053*	
C13	0.5108 (7)	0.7422 (6)	1.0267 (3)	0.0447 (13)	
H13	0.4561	0.7941	1.0652	0.054*	
C14	0.6464 (7)	0.5955 (6)	1.0504 (3)	0.0399 (12)	
H14	0.6846	0.5462	1.1040	0.048*	
C15	0.7235 (6)	0.5244 (5)	0.9898 (2)	0.0294 (10)	
C16	0.8857 (6)	0.3640 (5)	0.9178 (2)	0.0265 (9)	
C17	1.0300 (7)	0.2219 (5)	0.9025 (3)	0.0362 (11)	
H17A	1.0044	0.1368	0.9349	0.043*	
H17B	1.1595	0.2109	0.9203	0.043*	
C18	1.0314 (7)	0.2116 (5)	0.8160 (3)	0.0336 (10)	
H18A	1.1505	0.1298	0.8095	0.040*	
H18B	0.9214	0.1882	0.8028	0.040*	
N1	0.7582 (5)	0.5483 (4)	0.63286 (18)	0.0229 (7)	
N2	0.7352 (5)	0.6619 (4)	0.50395 (19)	0.0317 (8)	
H2	0.7239	0.7317	0.4612	0.038*	
N3	0.8577 (5)	0.6833 (4)	0.73877 (18)	0.0249 (8)	
H3B	0.8654	0.7008	0.7872	0.030*	
H3C	0.9790	0.6530	0.7222	0.030*	
N4	0.7730 (5)	0.4905 (4)	0.86430 (18)	0.0233 (8)	
N5	0.8614 (5)	0.3800 (4)	0.9931 (2)	0.0346 (9)	
H5	0.9212	0.3121	1.0359	0.042*	
N6	1.0196 (4)	0.3571 (4)	0.76028 (19)	0.0247 (8)	
H6A	1.0511	0.3406	0.7119	0.030*	
H6B	1.1082	0.3872	0.7783	0.030*	
N7	0.4435 (5)	0.5208 (4)	0.7462 (2)	0.0332 (8)	
O1	0.6072 (4)	0.3975 (3)	0.75714 (15)	0.0238 (6)	
O2	0.4853 (4)	0.6428 (3)	0.73689 (15)	0.0248 (6)	
O3	0.2770 (4)	0.5211 (3)	0.74554 (16)	0.0325 (7)	
O4	0.7163 (5)	0.8522 (4)	0.35574 (18)	0.0491 (9)	
H1O4	0.6480	0.8549	0.3158	0.059*	
H2O4	0.7584	0.9225	0.3391	0.059*	
O5	0.4947 (5)	0.8627 (4)	0.2253 (2)	0.0512 (9)	
H1O5	0.4095	0.9325	0.1902	0.061*	
H2O5	0.4738	0.7817	0.2302	0.061*	
O6	0.1492 (5)	0.9162 (4)	0.7010 (2)	0.0515 (9)	
H1O6	0.2360	0.8278	0.7051	0.062*	
H2O6	0.0865	0.9132	0.7425	0.062*	

### Atomic displacement parameters (Å^2^)

**Table:**

	*U*^11^	*U*^22^	*U*^33^	*U*^12^	*U*^13^	*U*^23^
Co1	0.0188 (3)	0.0192 (3)	0.0200 (3)	−0.0091 (2)	0.0009 (2)	−0.0046 (2)
Cl1	0.0477 (7)	0.0351 (7)	0.0390 (7)	−0.0174 (6)	−0.0086 (5)	−0.0009 (5)
C1	0.022 (2)	0.030 (2)	0.022 (2)	−0.0093 (19)	0.0009 (17)	−0.0119 (18)
C2	0.039 (3)	0.032 (2)	0.031 (2)	−0.017 (2)	0.005 (2)	−0.011 (2)
C3	0.050 (3)	0.036 (3)	0.048 (3)	−0.020 (2)	0.004 (2)	−0.022 (2)
C4	0.049 (3)	0.054 (3)	0.040 (3)	−0.027 (3)	0.005 (2)	−0.028 (3)
C5	0.037 (3)	0.059 (3)	0.023 (2)	−0.022 (2)	0.0043 (19)	−0.017 (2)
C6	0.021 (2)	0.037 (3)	0.027 (2)	−0.010 (2)	0.0001 (18)	−0.007 (2)
C7	0.028 (2)	0.028 (2)	0.020 (2)	−0.0139 (19)	0.0017 (17)	−0.0045 (18)
C8	0.048 (3)	0.028 (2)	0.032 (2)	−0.017 (2)	0.005 (2)	−0.001 (2)
C9	0.032 (2)	0.018 (2)	0.033 (2)	−0.0085 (19)	−0.0006 (19)	−0.0039 (18)
C10	0.024 (2)	0.029 (2)	0.025 (2)	−0.0147 (19)	0.0019 (17)	−0.0097 (18)
C11	0.036 (3)	0.030 (2)	0.026 (2)	−0.009 (2)	0.0018 (19)	−0.0075 (19)
C12	0.036 (3)	0.049 (3)	0.050 (3)	−0.012 (3)	0.011 (2)	−0.028 (3)
C13	0.046 (3)	0.069 (4)	0.037 (3)	−0.028 (3)	0.019 (2)	−0.036 (3)
C14	0.046 (3)	0.064 (4)	0.020 (2)	−0.030 (3)	0.007 (2)	−0.016 (2)
C15	0.036 (3)	0.039 (3)	0.021 (2)	−0.023 (2)	0.0021 (18)	−0.0062 (19)
C16	0.031 (2)	0.028 (2)	0.024 (2)	−0.018 (2)	−0.0026 (18)	−0.0012 (18)
C17	0.037 (3)	0.025 (2)	0.035 (3)	−0.006 (2)	−0.009 (2)	0.006 (2)
C18	0.032 (2)	0.023 (2)	0.041 (3)	−0.009 (2)	0.005 (2)	−0.003 (2)
N1	0.0239 (18)	0.0219 (18)	0.0241 (18)	−0.0094 (15)	0.0024 (14)	−0.0071 (15)
N2	0.041 (2)	0.032 (2)	0.0208 (18)	−0.0159 (18)	0.0025 (16)	−0.0005 (16)
N3	0.0266 (19)	0.0244 (19)	0.0246 (18)	−0.0107 (16)	0.0009 (14)	−0.0064 (15)
N4	0.0250 (19)	0.0222 (18)	0.0219 (17)	−0.0082 (16)	−0.0014 (14)	−0.0055 (15)
N5	0.041 (2)	0.037 (2)	0.0193 (18)	−0.0133 (19)	−0.0047 (16)	0.0021 (16)
N6	0.0179 (17)	0.0263 (19)	0.0310 (19)	−0.0096 (15)	0.0020 (14)	−0.0075 (15)
N7	0.0252 (12)	0.0359 (15)	0.043 (2)	−0.0149 (11)	0.0013 (16)	−0.0121 (18)
O1	0.0239 (13)	0.0269 (14)	0.0258 (14)	−0.0155 (10)	0.0026 (12)	−0.0062 (12)
O2	0.0180 (14)	0.0258 (15)	0.0295 (15)	−0.0068 (12)	0.0002 (12)	−0.0075 (13)
O3	0.0229 (14)	0.0463 (19)	0.0324 (16)	−0.0175 (14)	0.0022 (13)	−0.0103 (14)
O4	0.064 (2)	0.050 (2)	0.0339 (18)	−0.0329 (19)	−0.0067 (16)	0.0074 (16)
O5	0.050 (2)	0.037 (2)	0.064 (2)	−0.0182 (18)	−0.0146 (18)	−0.0055 (17)
O6	0.047 (2)	0.037 (2)	0.059 (2)	−0.0079 (17)	0.0078 (17)	−0.0037 (17)

### Geometric parameters (Å, º)

**Table:**

Co1—O1	1.928 (3)	C12—C13	1.379 (7)
Co1—O2	1.930 (3)	C12—H12	0.9300
Co1—N4	1.948 (3)	C13—C14	1.375 (7)
Co1—N6	1.949 (3)	C13—H13	0.9300
Co1—N1	1.953 (3)	C14—C15	1.392 (6)
Co1—N3	1.957 (3)	C14—H14	0.9300
C1—C2	1.388 (6)	C15—N5	1.388 (6)
C1—C6	1.400 (6)	C16—N4	1.337 (5)
C1—N1	1.412 (5)	C16—N5	1.349 (5)
C2—C3	1.383 (6)	C16—C17	1.483 (6)
C2—H2A	0.9300	C17—C18	1.521 (6)
C3—C4	1.404 (7)	C17—H17A	0.9700
C3—H3	0.9300	C17—H17B	0.9700
C4—C5	1.360 (7)	C18—N6	1.491 (5)
C4—H4	0.9300	C18—H18A	0.9700
C5—C6	1.399 (6)	C18—H18B	0.9700
C5—H5A	0.9300	N2—H2	0.8600
C6—N2	1.372 (5)	N3—H3B	0.9000
C7—N1	1.331 (5)	N3—H3C	0.9000
C7—N2	1.351 (5)	N5—H5	0.8600
C7—C8	1.484 (6)	N6—H6A	0.9000
C8—C9	1.504 (5)	N6—H6B	0.9000
C8—H8A	0.9700	N7—O3	1.233 (4)
C8—H8B	0.9700	N7—O2	1.319 (4)
C9—N3	1.496 (5)	N7—O1	1.327 (4)
C9—H9A	0.9700	O4—H1O4	0.8500
C9—H9B	0.9700	O4—H2O4	0.8496
C10—C11	1.383 (6)	O5—H1O5	0.8501
C10—C15	1.394 (5)	O5—H2O5	0.8500
C10—N4	1.419 (5)	O6—H1O6	0.8500
C11—C12	1.394 (6)	O6—H2O6	0.8499
C11—H11	0.9300		
			
O1—Co1—O2	68.37 (12)	C14—C13—C12	121.3 (4)
O1—Co1—N4	89.76 (12)	C14—C13—H13	119.3
O2—Co1—N4	92.05 (12)	C12—C13—H13	119.3
O1—Co1—N6	98.11 (13)	C13—C14—C15	116.5 (4)
O2—Co1—N6	166.39 (13)	C13—C14—H14	121.7
N4—Co1—N6	89.37 (14)	C15—C14—H14	121.7
O1—Co1—N1	91.42 (12)	N5—C15—C14	131.0 (4)
O2—Co1—N1	89.38 (12)	N5—C15—C10	106.1 (3)
N4—Co1—N1	178.40 (14)	C14—C15—C10	122.9 (4)
N6—Co1—N1	89.40 (13)	N4—C16—N5	111.3 (4)
O1—Co1—N3	166.29 (13)	N4—C16—C17	128.0 (4)
O2—Co1—N3	97.97 (13)	N5—C16—C17	120.6 (4)
N4—Co1—N3	89.66 (13)	C16—C17—C18	115.8 (3)
N6—Co1—N3	95.58 (14)	C16—C17—H17A	108.3
N1—Co1—N3	89.44 (13)	C18—C17—H17A	108.3
C2—C1—C6	120.0 (4)	C16—C17—H17B	108.3
C2—C1—N1	132.3 (4)	C18—C17—H17B	108.3
C6—C1—N1	107.7 (4)	H17A—C17—H17B	107.4
C3—C2—C1	117.8 (4)	N6—C18—C17	111.2 (3)
C3—C2—H2A	121.1	N6—C18—H18A	109.4
C1—C2—H2A	121.1	C17—C18—H18A	109.4
C2—C3—C4	121.4 (5)	N6—C18—H18B	109.4
C2—C3—H3	119.3	C17—C18—H18B	109.4
C4—C3—H3	119.3	H18A—C18—H18B	108.0
C5—C4—C3	121.5 (4)	C7—N1—C1	106.1 (3)
C5—C4—H4	119.2	C7—N1—Co1	125.7 (3)
C3—C4—H4	119.2	C1—N1—Co1	128.2 (3)
C4—C5—C6	117.2 (4)	C7—N2—C6	108.1 (3)
C4—C5—H5A	121.4	C7—N2—H2	125.9
C6—C5—H5A	121.4	C6—N2—H2	125.9
N2—C6—C5	131.4 (4)	C9—N3—Co1	112.9 (2)
N2—C6—C1	106.5 (3)	C9—N3—H3B	109.0
C5—C6—C1	122.0 (4)	Co1—N3—H3B	109.0
N1—C7—N2	111.6 (4)	C9—N3—H3C	109.0
N1—C7—C8	127.2 (3)	Co1—N3—H3C	109.0
N2—C7—C8	121.1 (4)	H3B—N3—H3C	107.8
C7—C8—C9	116.8 (4)	C16—N4—C10	106.1 (3)
C7—C8—H8A	108.1	C16—N4—Co1	125.6 (3)
C9—C8—H8A	108.1	C10—N4—Co1	128.3 (3)
C7—C8—H8B	108.1	C16—N5—C15	108.5 (3)
C9—C8—H8B	108.1	C16—N5—H5	125.8
H8A—C8—H8B	107.3	C15—N5—H5	125.8
N3—C9—C8	111.6 (3)	C18—N6—Co1	113.3 (2)
N3—C9—H9A	109.3	C18—N6—H6A	108.9
C8—C9—H9A	109.3	Co1—N6—H6A	108.9
N3—C9—H9B	109.3	C18—N6—H6B	108.9
C8—C9—H9B	109.3	Co1—N6—H6B	108.9
H9A—C9—H9B	108.0	H6A—N6—H6B	107.7
C11—C10—C15	119.7 (4)	O3—N7—O2	125.0 (4)
C11—C10—N4	132.3 (4)	O3—N7—O1	125.0 (4)
C15—C10—N4	108.0 (4)	O2—N7—O1	110.0 (3)
C10—C11—C12	117.4 (4)	N7—O1—Co1	90.7 (2)
C10—C11—H11	121.3	N7—O2—Co1	90.9 (2)
C12—C11—H11	121.3	H1O4—O4—H2O4	104.9
C13—C12—C11	122.1 (5)	H1O5—O5—H2O5	107.7
C13—C12—H12	118.9	H1O6—O6—H2O6	107.7
C11—C12—H12	118.9		

### Hydrogen-bond geometry (Å, º)

**Table:**

*D*—H···*A*	*D*—H	H···*A*	*D*···*A*	*D*—H···*A*
N2—H2···O4	0.86	1.91	2.757 (5)	168
N3—H3*B*···Cl1^i^	0.90	2.56	3.322 (3)	143
N3—H3*C*···O3^ii^	0.90	2.08	2.897 (4)	150
N5—H5···Cl1^iii^	0.86	2.29	3.148 (4)	174
N6—H6*A*···O4^i^	0.90	2.54	3.299 (5)	142
N6—H6*B*···O3^ii^	0.90	2.12	2.897 (4)	145
O4—H1*O*4···O5	0.85	1.92	2.766 (4)	179
O4—H2*O*4···O6^iv^	0.85	1.93	2.781 (5)	179
O5—H1*O*5···Cl1^v^	0.85	2.41	3.189 (3)	153
O5—H2*O*5···O1^vi^	0.85	2.03	2.872 (4)	174
O6—H1*O*6···O2	0.85	2.01	2.828 (4)	162
O6—H2*O*6···Cl1^vi^	0.85	2.34	3.192 (4)	176

Symmetry codes: (i) −*x*+2, −*y*+1, −*z*+1; (ii) *x*+1, *y*, *z*; (iii) *x*, *y*, *z*+1; (iv) −*x*+1, −*y*+2, −*z*+1; (v) *x*−1, *y*+1, *z*; (vi) −*x*+1, −*y*+1, −*z*+1.
